# Influence of Fibers on the Flexural Performance and Damage Evolution Behavior of Coal-Based Solid Waste Grouting Material

**DOI:** 10.3390/ma19194254

**Published:** 2026-10-08

**Authors:** Yachao Guo, Yitong Wang, Junmeng Li, Yanli Huang, Zuo Sun, Jiachen Liu, Ding Liu

**Affiliations:** 1 School of Mines, China University of Mining & Technology, Xuzhou 221116, China; 2 Key Laboratory of Xinjiang Coal Resources Green Mining, Xinjiang Institute of Engineering, Ministry of Education, Urumqi 830023, China; 3 Xinjiang Engineering Research Center of Green Intelligent Coal Mining, Xinjiang Institute of Engineering, Urumqi 830023, China; 4 State Key Laboratory of Safe Mining of Deep Coal and Environmental Protection, Huainan Mining (Group) Co., Ltd., Huainan 232000, China

**Keywords:** coal-based solid waste, grouting materials, fiber toughening, damage evolution

## Abstract

To address the engineering challenges associated with the high brittleness and poor crack resistance of coal-based solid waste grouting materials, this study systematically investigates the flexural performance and damage evolution characteristics of the material by incorporating various types and amounts of fibers into a matrix composed of coal gangue, fly ash, Portland cement, and sulfoaluminate cement. Macroscopic mechanical testing, digital image correlation (DIC) technology, and scanning electron microscopy (SEM) are employed to reveal the mechanisms by which fibers influence the grouting material. The results demonstrate that fiber type and dosage significantly affect the flexural strength and damage evolution behavior of the material. Among them, basalt fiber exhibits the most pronounced improvement in flexural strength at a dosage of 0.4%, achieving a 28-day flexural strength of 6.33 MPa, which is 73.9% higher than that of the control group. Similarly, glass fiber shows a flexural strength increase of 63.18% at a dosage of 0.8%. The fibers enhance the material’s ductility and promote the uniformity of the failure process through mechanisms such as crack bridging, energy dissipation, and optimized interfacial structure. Furthermore, joint analysis using DIC and acoustic emission (AE) techniques reveals that when the fiber dosage reaches 0.6%, the proportion of shear cracks during material failure significantly increases (12.78–16.13%), indicating that fibers improve the uniformity of strain distribution under loading, delay the propagation of the main crack, and thereby enhance the overall crack resistance of the material. These findings provide theoretical and technical support for the development of high-toughness and high-performance coal-based solid waste grouting materials.

## 1. Introduction

In large-scale underground engineering activities such as coal mining, underground gasification, deep roadway construction, and goaf treatment, complex geotechnical challenges such as high in situ stress, intense rock mass disturbance, and significant water-rock interactions are increasingly becoming prominent [1,2]. These issues directly lead to a reduction in foundation bearing capacity, highly developed rock fractures, and a marked increase in leakage risks, posing severe challenges to engineering stability and safety [3,4]. Grouting technology, which involves injecting specific grout slurry into cracks or pores within rock and soil masses to achieve the bonding and reinforcement of fractured rock, is a key technology for ensuring the long-term stability of underground engineering [5]. Nevertheless, traditional cement-based grouting materials often suffer from inherent disadvantages such as large particle size, poor injectability, and long setting time, and their production process is energy-intensive and accompanied by high carbon emissions. In addition, although chemical grouting materials exhibit excellent performance, their high cost and potential environmental pollution risks make them difficult to widely promote and apply in large-scale engineering projects [6]. Therefore, the development of a new type of grouting material with low cost, high performance, and environmental friendliness has become an urgent research frontier and a hot topic.

Coal mining and coal cleaning processes generate coal gangue, fly ash, and other coal-based solid wastes. The massive accumulation of these wastes not only occupies valuable land resources but also poses a serious threat to soil and groundwater environments through leaching [7]. Notably, these wastes contain abundant active components such as SiO_2_ and Al_2_O_3_, which provide a promising raw material for the preparation of high-performance grouting materials [8]. In recent years, scholars both domestically and internationally have made active explorations into the application of coal-based solid wastes in grouting materials. For example, Ou Yang et al. systematically studied the mechanical properties and durability of polymer grouting materials based on coal gangue and fly ash, confirming that their compressive strength can reach over 30 MPa and that they exhibit excellent corrosion resistance, offering strong evidence for the engineering feasibility of solid waste-based grouting materials [9]. Sasui et al. focused on the influence of activators on the hydration products of the material, effectively optimizing the early strength and workability of the material through the composite use of alkaline activators [10]. Kampragkou, P et al. found that the incorporation of biobased additives exerted a positive influence on the volume stability, hygrothermal performance, and post-cracking behavior (in certain cases) of mortar, thereby improving these properties compared to the conventional control group (i.e., materials without biobased additives). However, in some scenarios, the biobased additives notably reduced the flexural, compressive, and tensile strengths of the mortar [11]. Pan et al. designed a paste-type grouting material based on coal gangue and applied it for backfilling in goafs, effectively controlling surface subsidence [12]. Despite these advancements, the inherent brittleness and low toughness of such solid waste-based materials remain the core bottlenecks limiting their large-scale engineering application. Under load, these materials tend to undergo brittle fracture immediately upon reaching their peak strength, exhibiting extremely low fracture energy and strain energy absorption capacity [13]. As a result, they are prone to secondary cracking under dynamic loads or in complex stress conditions, which may even lead to failure of the reinforcement system.

Fibers, by forming a “micro-reinforcement” three-dimensional random network within the matrix, can effectively bridge crack tips and inhibit crack propagation, thereby significantly enhancing the material’s impact resistance and crack resistance. This has become an effective approach for improving the material’s toughness [14,15]. The core toughening mechanism lies in the fact that when the material undergoes deformation under stress and microcracks are formed, high-modulus fibers span across the crack surface and transfer the stress through interfacial adhesion between the fibers and the matrix, which is referred to as the “bridging effect” [16,17]. As the crack continues to propagate, the fibers undergo slippage until they are pulled out, a process that consumes a significant amount of mechanical energy and greatly enhances the material’s fracture toughness [18]. Currently, there have been extensive studies on the strengthening effects of various fibers, such as steel fibers, polypropylene fibers, and basalt fibers, on cement-based materials, and their effectiveness in improving impact performance and suppressing plastic shrinkage cracks has been well confirmed [19,20]. For instance, Zhao et al. [21] employed uniaxial compressive strength and acoustic emission monitoring techniques to investigate the effects of polyacrylonitrile fiber on the mechanical properties, AE response characteristics, and precursors of damage in CTB mortars with varying cement-to-sand ratios. Similarly, Yin et al. conducted uniaxial compression tests to examine the impact of polypropylene fiber on the mechanical behavior, failure patterns, and damage evolution of sulfur-containing CTB mortars [22]. However, when applying existing fiber reinforcement theories directly to the complex system of coal-based solid waste grouting materials, it remains unclear how different types of fibers regulate the macroscopic mechanical behavior and damage evolution process by altering the material’s microstructure. Are there fundamental differences in their distinct toughening mechanisms? These key scientific questions still lack systematic research.

Given this context, this paper takes coal gangue, fly ash, and blended cement as raw materials and introduces fiber modification to systematically investigate the effects of different types and dosages of fibers on the flexural performance and damage evolution behavior of the material. By employing a combination of macroscopic mechanical property tests, DIC technology, SEM, and other multi-method approaches, the study aims to establish the structure-property relationship between fiber parameters (type and content) and the material’s macroscopic mechanical response and damage evolution behavior, and to reveal the distinct toughening mechanisms of different fibers within coal-based solid waste grouting materials. The research outcomes provide a solid theoretical foundation and technological pathway for the development of green coal-based solid waste grouting materials with high toughness and long service life.

## 2. Materials and Methods

### 2.1. Raw Materials and Physicochemical Properties

**Figure 1:**
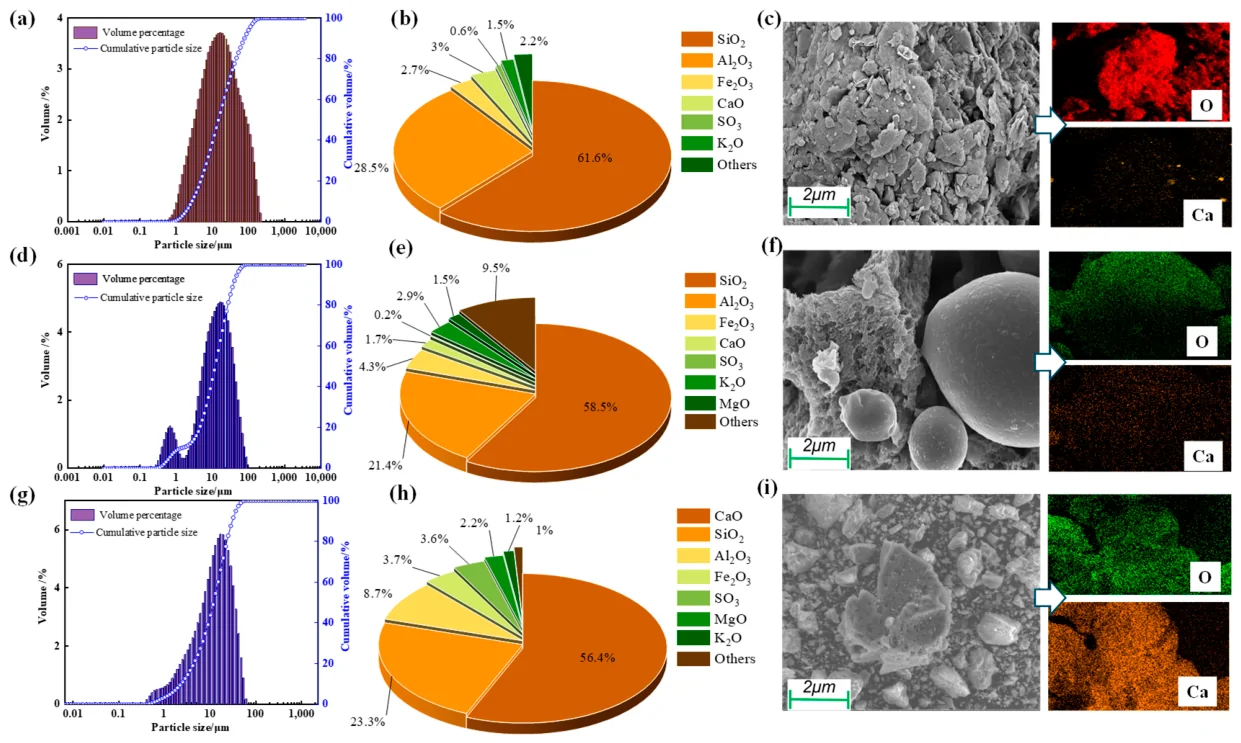
Particle size, chemical composition, and surface morphology of raw materials. (**a–c**): Coal gangue; (**d–f**): Fly ash; (**g–i**): Cement.

**Coal Gangue:** The coal gangue used in the experiment was initially crushed by a jaw crusher and subsequently ground using a ball mill, and finally sieved through a 325-mesh square aperture sieve. Its particle size distribution mainly falls within the range of 0.6 to 200 μm. The primary chemical composition of the coal gangue was analyzed by the PANalytical Axios series X-ray Fluorescence Spectrometer (X-ray Fluorescence Spectrometer, XRF), manufactured by PANalytical in Almelo, The Netherlands, revealing that it predominantly consists of oxides such as SiO_2_ (61.55%), Al_2_O_3_ (28.46%), Fe_2_O_3_ (2.71%), and CaO (2.98%). X-ray Diffraction (XRD) analysis indicates that the mineralogical composition is mainly composed of quartz, muscovite, and kaolinite, as illustrated in Figure 1.

**Fly ash**: The fly ash used in the experiment has a particle size distribution ranging from 0.3 to 100 μm. Its main composition is similar to that of coal gangue, with SiO_2_ (58.45%), Al_2_O_3_ (21.4%), CaO (1.67%), and Fe_2_O_3_ (4.31%) as the primary components. In addition, its mineral phases include quartz, mullite, and hematite.

**Cement:** The cement used in this experiment includes Portland cement and sulfoaluminate cement. The particle size distribution of Portland cement ranges from 0.6 to 100 μm, and its main mineral composition consists of tricalcium silicate (C_3_S), dicalcium silicate (C_2_S), tricalcium aluminate (C_3_A), and tetracalcium aluminoferrite (C_4_AF). Its main chemical composition includes oxides such as CaO (56.37%), Al_2_O_3_ (8.71%), SO_3_ (3.61%), and SiO_2_ (23.31%). For sulfoaluminate cement, the main mineral composition is anhydrous calcium sulfoaluminate (C_4_A_3_S), calcium sulfate (CaSO_4_), and dicalcium silicate (C_2_S), with its particle size distribution ranging from 0.5 to 200 μm. The main chemical composition includes oxides such as CaO (41.61%), Al_2_O_3_ (29.68%), SO_3_ (20.59%), and SiO_2_ (6.29%).

**Superplasticizer**: The superplasticizer used in the experiment is a polycarboxylate high-performance superplasticizer with a water reduction rate of 45% and an air content of 2.5%.

**Fibers**: Four types of high-performance fibers were selected as reinforcing agents. Glass fiber (GL) is known for its high elastic modulus (greater than 85 GPa) and low density (2.699 g/cm^3^). It has a length of 12 mm and a diameter of up to 17.4 μm, but its elongation at break does not exceed 2.5%. Basalt fiber (BA) has a high elastic modulus of up to 100 GPa and a density of 3.05 g/cm^3^, with an elongation at break controlled below 4%, and the fiber length is also 12 mm. Coated copper micro-steel fiber (ST) has a density of 7.9 g/cm^3^, an elongation at break of over 20%, a length of 12 mm, and a diameter of 0.2 mm, effectively improving the material’s tensile and impact resistance. Polyvinyl alcohol (PVA) fiber has an elastic modulus of 40 GPa, a density of 1.305 g/cm^3^, a length of 12 mm, and an elongation at break as high as 30%, with a diameter of 15.3 μm. The core advantage of PVA fiber lies in its excellent wear resistance and acid-base resistance.

### 2.2. Test Mix Design and Testing Methods

Based on the optimized mix ratio from previous preliminary experiments (coal gangue powder: fly ash: cement = 5:1:4, water-cement ratio of 0.4, superplasticizer dosage of 1.0%, and the ratio of Portland cement to sulfoaluminate cement = 6:4) [20], this study systematically investigates the effects of fiber type and dosage on the performance of grouting materials. The ratio of Portland cement to sulfoaluminate cement is fixed at 6:4, as this mix proportion can meet the requirements for material strength and workability in engineering applications. Four typical fibers—BA, GL, PVA, and ST—are selected to examine their reinforcing effects under fiber dosages of 0.2%, 0.4%, 0.6%, 0.8%, and 1.0%. A fiber-free group is used as the control group. It is worth noting that all the measurements in this study are based on mass ratio.

The specimen preparation process is as follows: According to the experimental design, the fibers, coal gangue, fly ash, and cement were first added into a mixer and pre-blended for 60 s to ensure preliminary uniform dispersion of the components [23]. Subsequently, the well-mixed dry materials were combined with water (containing a water-reducing agent) and thoroughly homogenized to form a homogeneous slurry, which was immediately poured into triplex plastic molds with dimensions of 160 mm × 40 mm × 40 mm. To guarantee the reliability and representativeness of the test results, three replicate specimens were prepared for each group of mix proportions. After the specimens were cast and allowed to set at room temperature for 24 h, they were demolded once the slurry had reached initial setting. Finally, the specimens were transferred to a standard curing room (temperature 20 ± 2 °C, relative humidity 95 ± 2%) for 28 days of aging.

**Figure 2:**
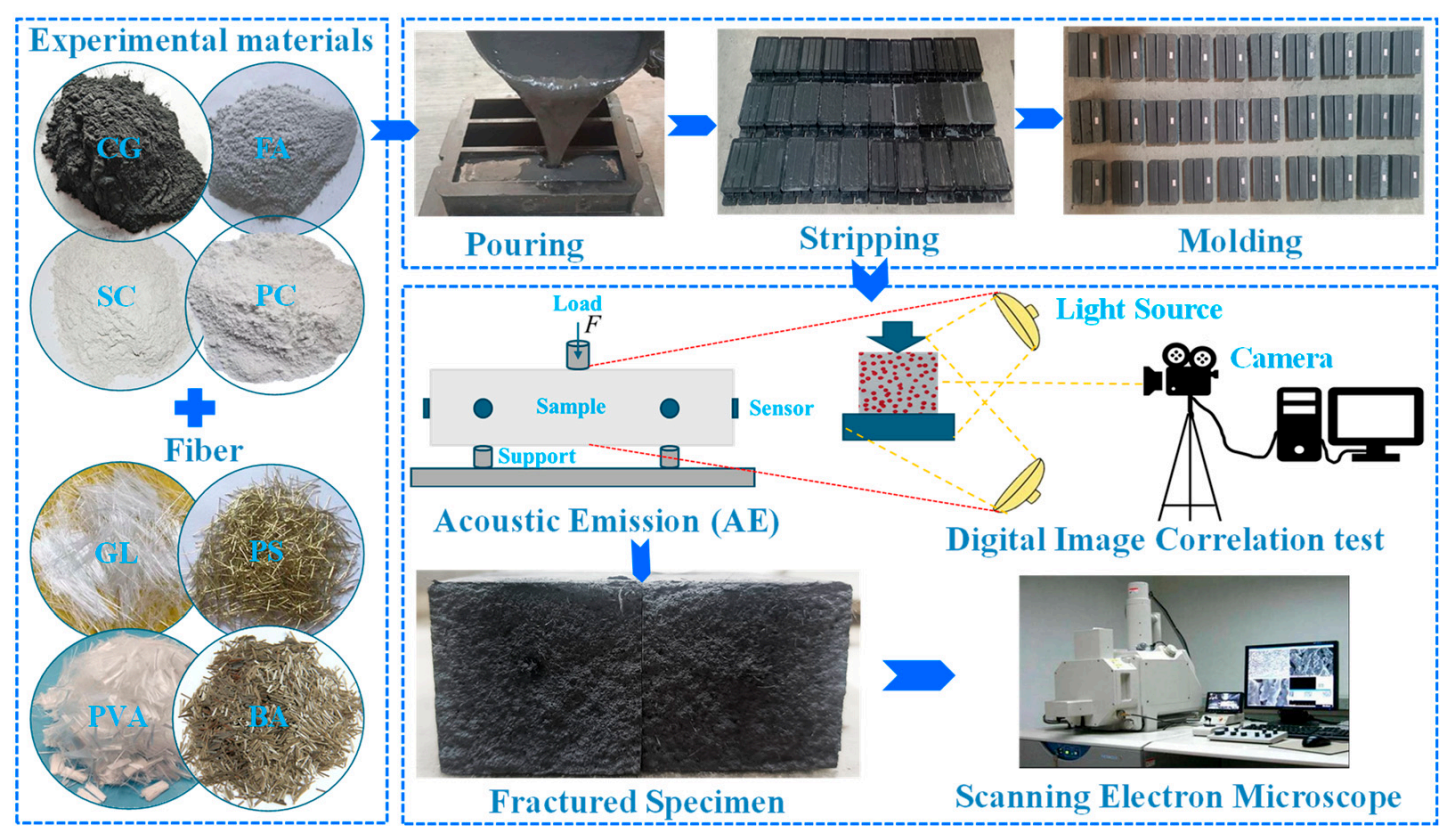
Sample preparation process and testing flowchart.

The flexural strength test was performed on cured specimens according to the reference method using a WDW-300 electric hydraulic servo universal testing machine at a loading rate of 1 mm/min [14,15]. The test is stopped when the elongation at break reaches 70% of the peak load, and the stress–strain curves are recorded. The flexural strength is calculated as the average of the results from the three specimens. In addition, the acoustic emission characteristics, flexural deformation behavior, and microstructure of the specimen during failure are characterized and analyzed. The specific testing process is illustrated in Figure 2.

During the microstructural analysis of the material, samples of different ages are first thoroughly dried. Then, small pieces with natural fracture surfaces are selected and mounted on the sample holder with the fracture face facing upward. After being subjected to gold coating treatment, the internal microstructure of the material is observed using a Quanta 250 SEM at magnification rates ranging from 1000 to 10,000 times.

### 2.3. Material Damage Characterization Methods

#### 2.3.1. DIC Technology Analysis Method

**Figure 3:**
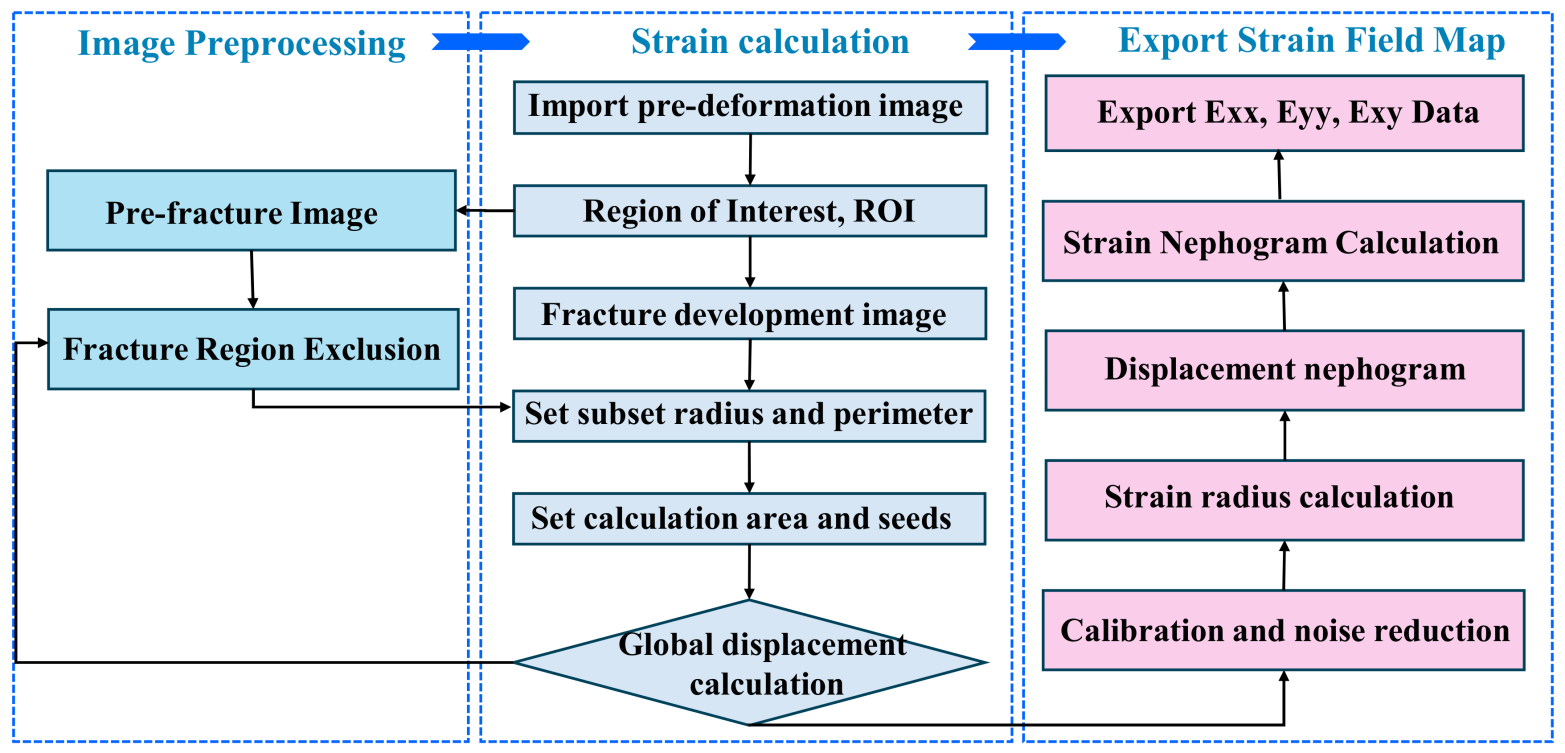
Processing flow of the DIC method.

The DIC technique is employed for analysis. DIC is a non-contact optical measurement technique that enables full-field strain measurement and deformation analysis by tracking the topographical changes on the surface of materials. With its high precision and non-destructive nature, DIC is widely employed for investigating the deformation behavior and crack evolution paths of materials under mechanical loading. During the sample preparation stage, the surface of the specimen is first finely ground to ensure flatness. Subsequently, a white base coat and red spots are sequentially sprayed to create an artificial speckle pattern. This pattern is characterized by strong randomness and high contrast, making it suitable for high-precision image analysis [24]. During the testing process, under the assistance of an LED light source, a high-speed camera continuously captures the video stream of the specimen’s bending deformation process. The original images are first calibrated using the DIC system and then processed with dedicated analysis software. The sampling frequency is set to 20 Hz, ultimately obtaining the full-field strain distribution on the specimen’s surface as well as the dynamic evolution process of crack initiation and propagation. After the completion of the experiments, one representative sample from each group was selected for processing and analysis. The detailed processing flow is shown in Figure 3.

#### 2.3.2. AE Technology and Analysis

To analyze the influence of fiber addition on the bending and fracture process of the specimens, the AE technique is used for dynamic monitoring of the damage process. AE is a technique that utilizes the elastic wave signals released during the formation and propagation of micro-cracks within materials to study internal damage mechanisms. It is primarily used for real-time monitoring of crack initiation, propagation, and failure processes in materials subjected to mechanical loading, offering advantages of high sensitivity and non-destructiveness. The test employs the PCI-2 system for synchronized measurements. AE sensors are symmetrically arranged on the side and back surfaces of the specimen to avoid differences in signal propagation paths. The sensors are fixed using coupling agents to ensure signal reliability. The RA-AF method is used for comprehensive characterization of crack damage evolution, where RA is a characteristic value reflecting the rise time of the AE waveform, and AF represents the average frequency [25]. The calculation methods for RA and AF are as follows:

(1) $$RA=\frac{RT}{A}$$

(2) $$AF=\frac{C}{DT}$$

where RT is the rise time of the acoustic emission event, A is the amplitude; C is the number of acoustic emission counts, and DT is the duration. The study focuses on the proportion of cracks during the material fracture process. According to the literature, the ratio of the maximum AF to the maximum RA, denoted as *k*, is used as the crack classification threshold. When *AF*/*RA* > *k*, the event is defined as a tensile crack event; when *AF*/*RA* < *k*, it is defined as a shear crack event [15].

## 3. Results and Discussion

### 3.1. Influence of Fiber Type and Content on the Flexural Performance of the Material

**Figure 4:**
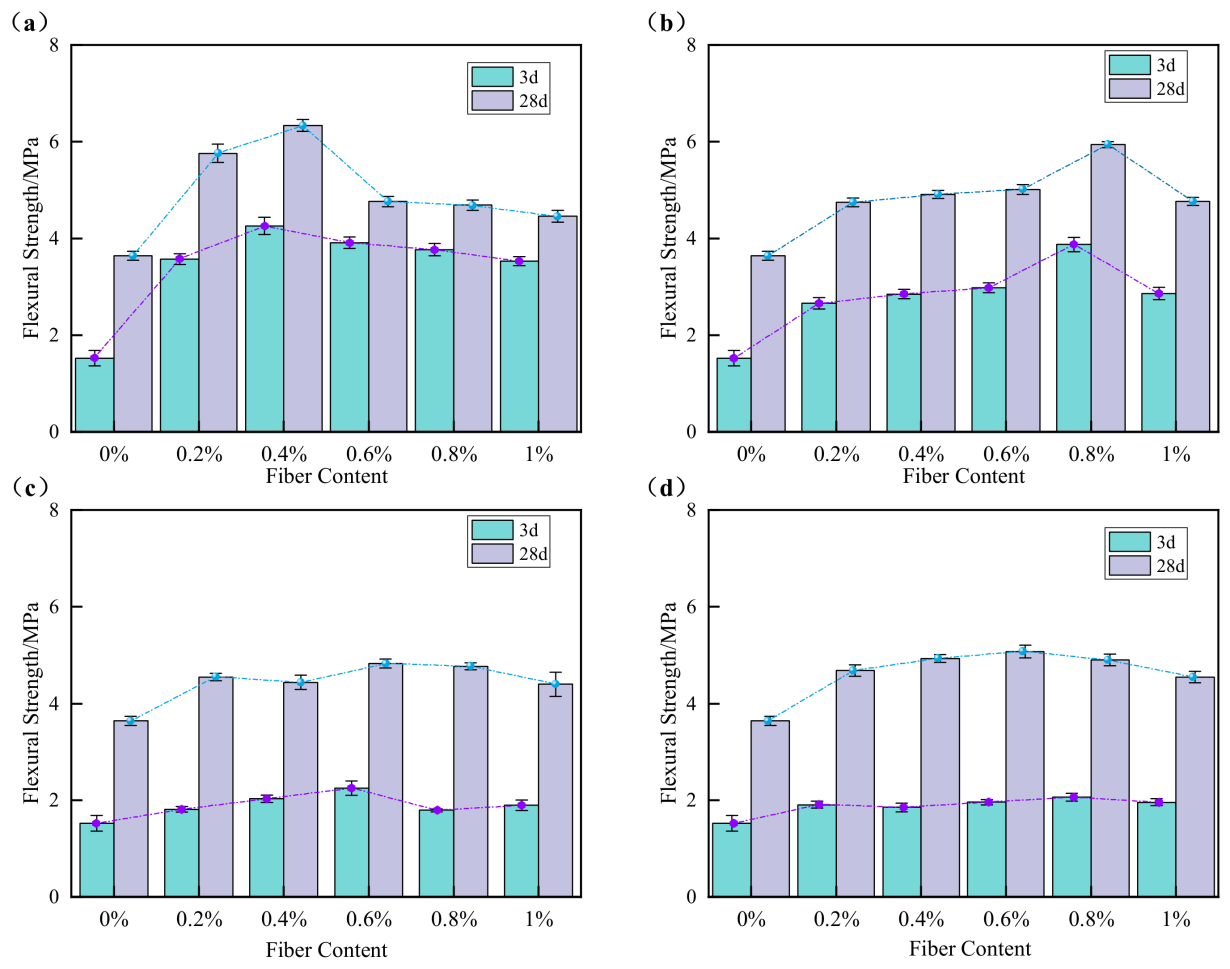
Influence of fiber type and content on the flexural strength of the material: (**a**) BA; (**b**) GL; (**c**) PVA; (**d**) ST.

Figure 4 shows the influence of fiber type and content on the flexural strength of the material. The flexural strength of the material generally exhibits an upward trend with increasing fiber content, followed by a decline. The basalt fiber demonstrates the best flexural strengthening effect at a content of 0.4%, with a 28-day flexural strength of up to 6.33 MPa, representing an increase of 73.9% compared to the control group. This is mainly due to its high elastic modulus and good bonding between the fiber and the matrix, which effectively inhibits the propagation of microcracks through bridging action. However, when the fiber content exceeds 0.6%, fiber agglomeration becomes pronounced, forming new weak interfaces and leading to a decrease in strength. The glass fiber achieves the maximum 28-day flexural strength of 5.94 MPa at a content of 0.8%, with an increase of 63.18%, attributed to its high tensile strength and stability. Both PVA fiber and copper-coated micro-steel fiber show optimal strengthening effects at 0.6% content, with flexural strengths of 4.9 MPa and 4.82 MPa, respectively. This is because the hydrophilicity of PVA fiber facilitates its bonding with the cement matrix [26] while steel fibers mainly provide toughening effects through their high strength. However, their relatively high density leads to poor dispersion in the slurry, causing them to accumulate at the bottom of the specimen and affecting the overall material performance. In conclusion, basalt fiber and glass fiber show excellent flexural strengthening potential in coal-based solid waste grout materials, and their optimal fiber content range (0.4–0.8%) provides clear guidance for engineering applications.

### 3.2. Surface Damage Patterns of F-SWG Materials with Different Fiber Types

**Figure 5:**
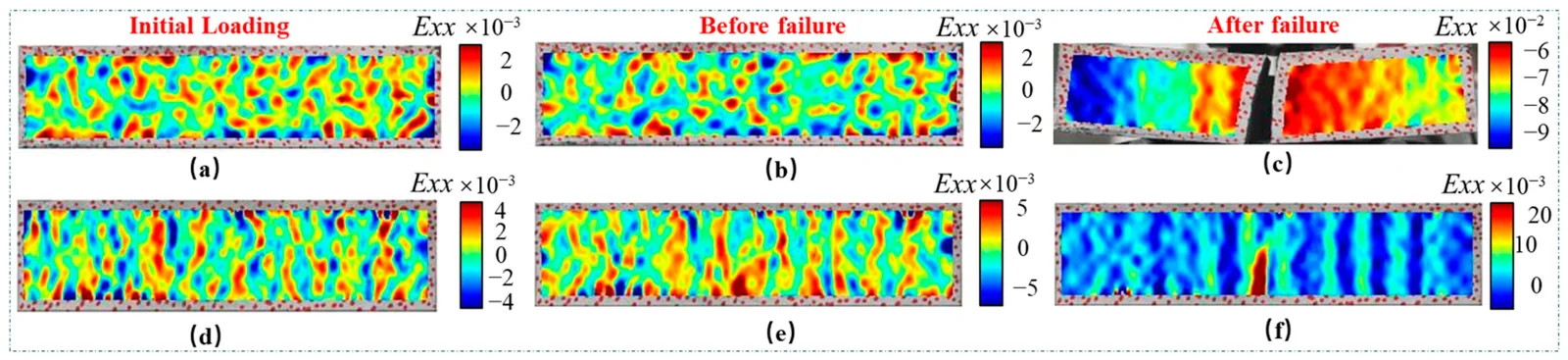
Horizontal strain distribution cloud images at different loading stages: (**a**–**c**) are FB = 0%; (**d**–**f**) are BA = 0.6%.

Figure 5 shows the surface damage patterns of F-SWG materials with different fiber types at various loading stages. In the early loading stage, the strain distribution cloud image of the specimen shows alternating red and blue regions, with relatively low overall strain. As loading continues, the surface cloud image remains relatively uniform, but the red region at the bottom becomes more pronounced, indicating that even before reaching the load-bearing limit, inherent non-uniformities such as uneven aggregate distribution and inconsistent hydration products have already caused localized stress concentration and micro-damage initiation. With further loading, several concentrated red regions at the bottom gradually merge into one, forming a red “flame” shaped area of stress concentration, which then extends upward and eventually leads to specimen failure. Comparison shows that after fiber incorporation, the failed specimens do not form obvious through-going cracks, suggesting that the fibers effectively improve the flexural performance of the material.

**Figure 6:**
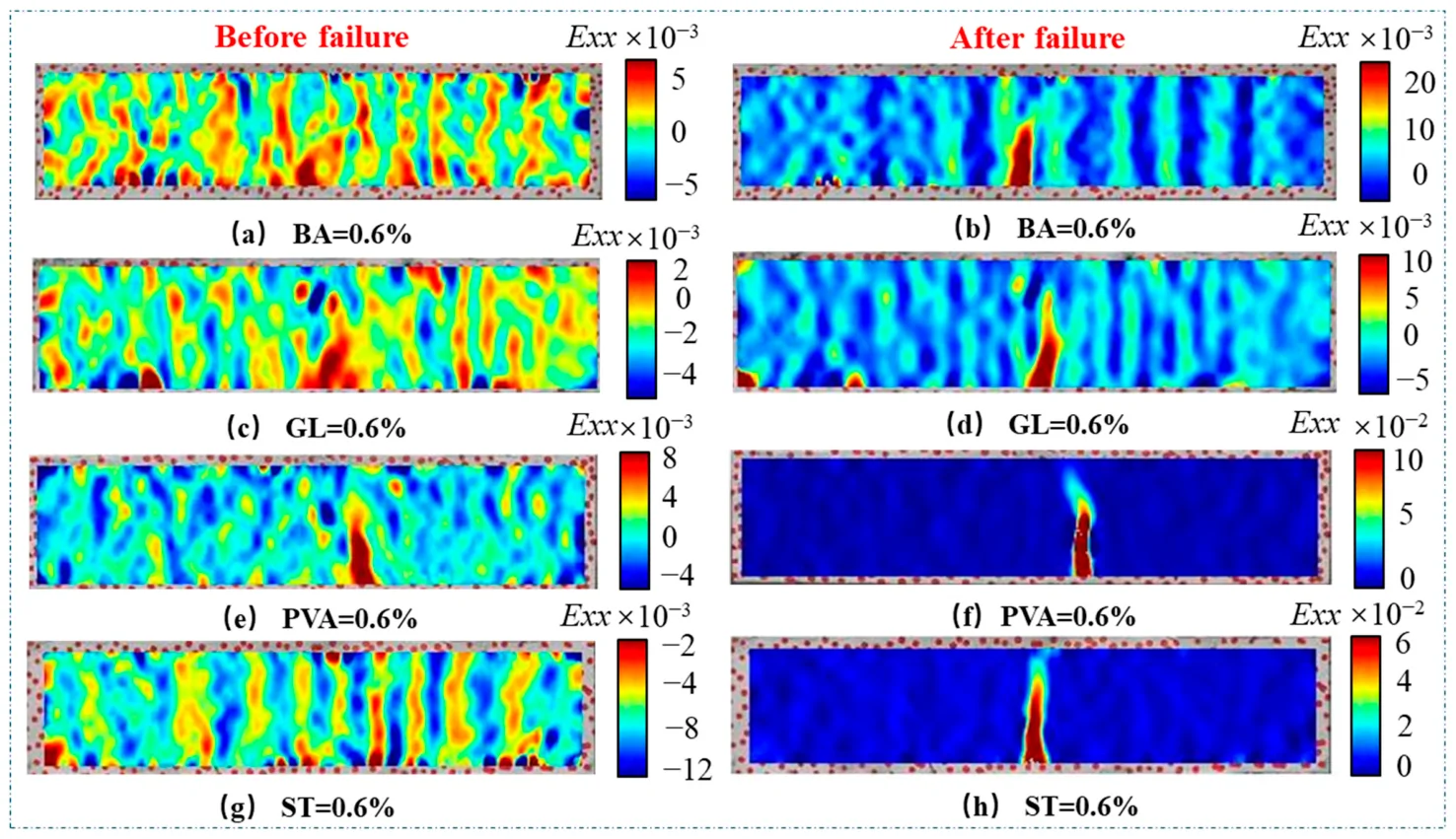
Horizontal strain distribution cloud images (before and after failure) for different fiber types at 0.6% reinforcement ratio: (**a**,**b**) BA; (**c**,**d**) GL; (**e**,**f**) PVA; (**g**,**h**) ST.

As shown in Figure 6, for both basalt fiber and glass fiber specimens, the high strain regions are diffusely distributed before failure. After failure, the basalt fiber specimens exhibit a significant bridging effect, with the main crack propagation path being irregular and tortuous. In contrast, the high strain regions in glass fiber specimens are slightly less extensive and more concentrated compared to those in basalt fiber specimens, and the crack width after failure is larger. This indicates that the basalt fibers, due to their high elastic modulus and excellent interfacial bonding performance, effectively disperse localized stresses and inhibit the aggregation and directional propagation of microcracks, thereby delaying the concentration process of damage. Similarly, the copper-coated micro steel fiber and PVA fiber specimens also show diffused strain distributions prior to failure. However, the steel fibers, due to their high density, tend to accumulate at the bottom of the specimen, leading to more localized strain concentration. On the other hand, PVA fibers, with their lower elastic modulus, have a limited effect on reducing strain concentration, suggesting that the mechanical properties and distribution state of the fibers determine the energy dissipation capacity and overall toughness of the material after failure.

### 3.3. AE Characteristics of F-SWG Materials Under Different Fiber Types

#### 3.3.1. Time-Stress Curves and AE Energy Features

**Figure 7:**
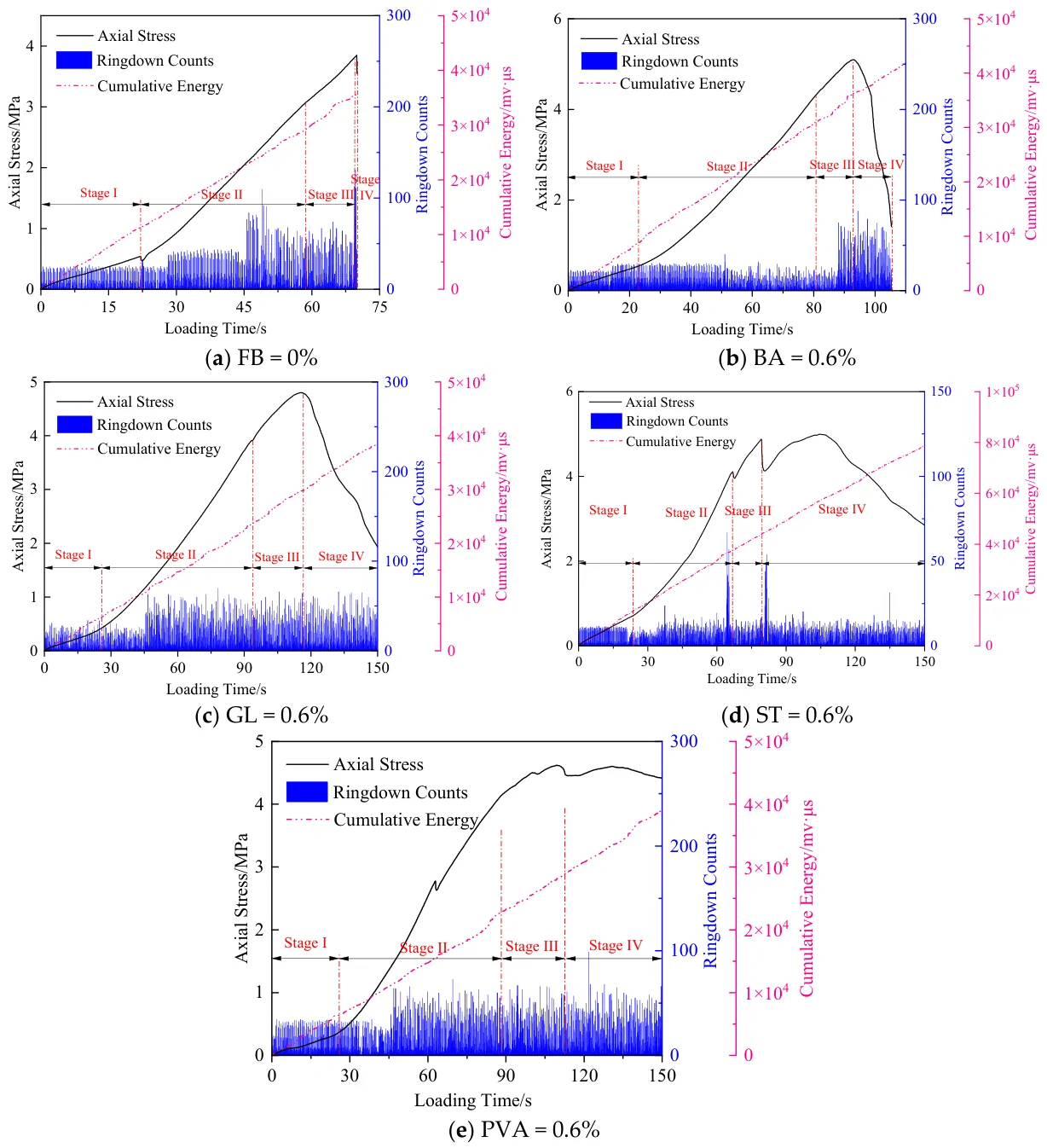
Influence of fiber types on stress-ringing count-accumulated energy relationship: (**a**) FB = 0%; (**b**) BA = 0.6%; (**c**) GL = 0.6%; (**d**) ST = 0.6%; (**e**) PVA = 0.6%.

Figure 7 compares the time-stress curves and acoustic emission hit counts of specimens with and without fiber addition, under different fiber types. The analysis shows that the control group without fiber addition exhibits typical brittle characteristics, with its time-stress curve experiencing an abrupt fracture immediately after reaching the peak stress (approximately 3.85 MPa), and the residual stress being zero. In sync with this process, the hit count of the acoustic emission signal shows a steep rise, indicating that the material’s failure process occurs within a very short time period, with energy release and damage events highly concentrated. The introduction of fibers significantly alters this evolutionary pattern. Taking basalt fiber as an example, its time-stress curve is no longer smooth during the ascending stage but instead shows a series of small, fluctuating “stress steps.” This phenomenon is highly synchronized with the acoustic emission signal, which exhibits a marked increase in hit counts at the early stages of loading, and maintains a high-frequency oscillation throughout the loading process, rather than experiencing a concentrated burst before failure. The glass fiber group shows a similar trend, but with a slightly lower overall activity and fluctuation amplitude in hit counts, reflecting its relatively lower interfacial bonding efficiency. In contrast, the PVA and steel fiber groups display less obvious fluctuations in the stress curves, and their acoustic emission hit counts also remain relatively low before reaching the peak load, indicating their limited ability to suppress micro-scale crack propagation and trigger continuous stress redistribution. However, the peak load is increased in all fiber-reinforced specimens.

**Figure 8:**
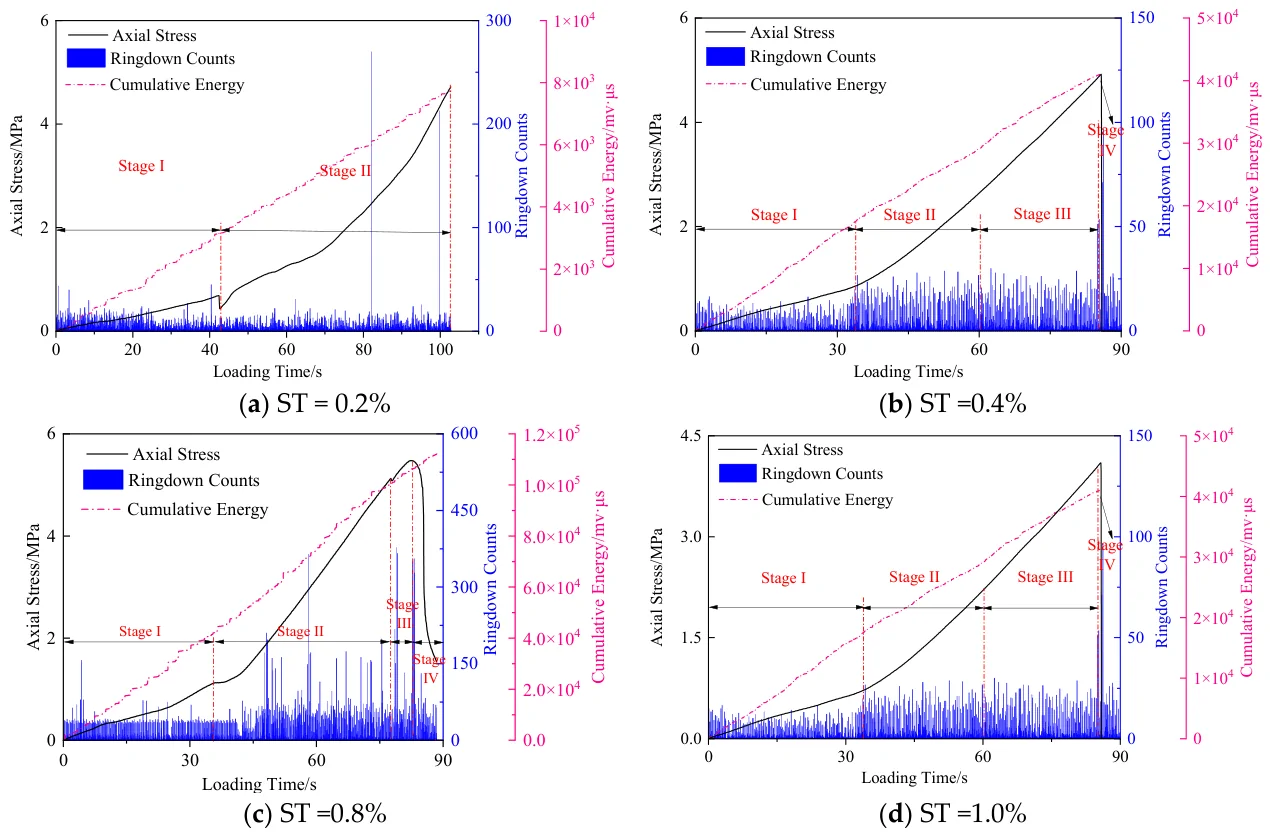
Impact of copper-coated micro-steel fiber content on the stress-ringing count-accumulated energy relationship: (**a**) ST = 0.2%; (**b**) ST = 0.4%; (**c**) ST = 0.8%; (**d**) ST = 1.0%.

As illustrated in Figure 8, the AE response of the material under different contents of copper-coated micro-steel fibers is presented. It can be observed that with the increase in the content of copper-coated micro-steel fibers, the curve begins to exhibit a post-peak stage, indicating an improvement in the material’s ductility. Concurrently, when the content of copper-coated micro-steel fibers reaches 0.8% and 1.0%, the number of AE events during sample failure becomes significantly denser. This suggests that a more extensive and dense fiber bridging network has been formed within the matrix, which enhances the material’s ability to detect and restrain microcracks. Consequently, more pronounced stress redistribution and more frequent and intense AE events are triggered.

#### 3.3.2. Characterization of Tensile and Shear Cracks in the Specimens

**Figure 9:**
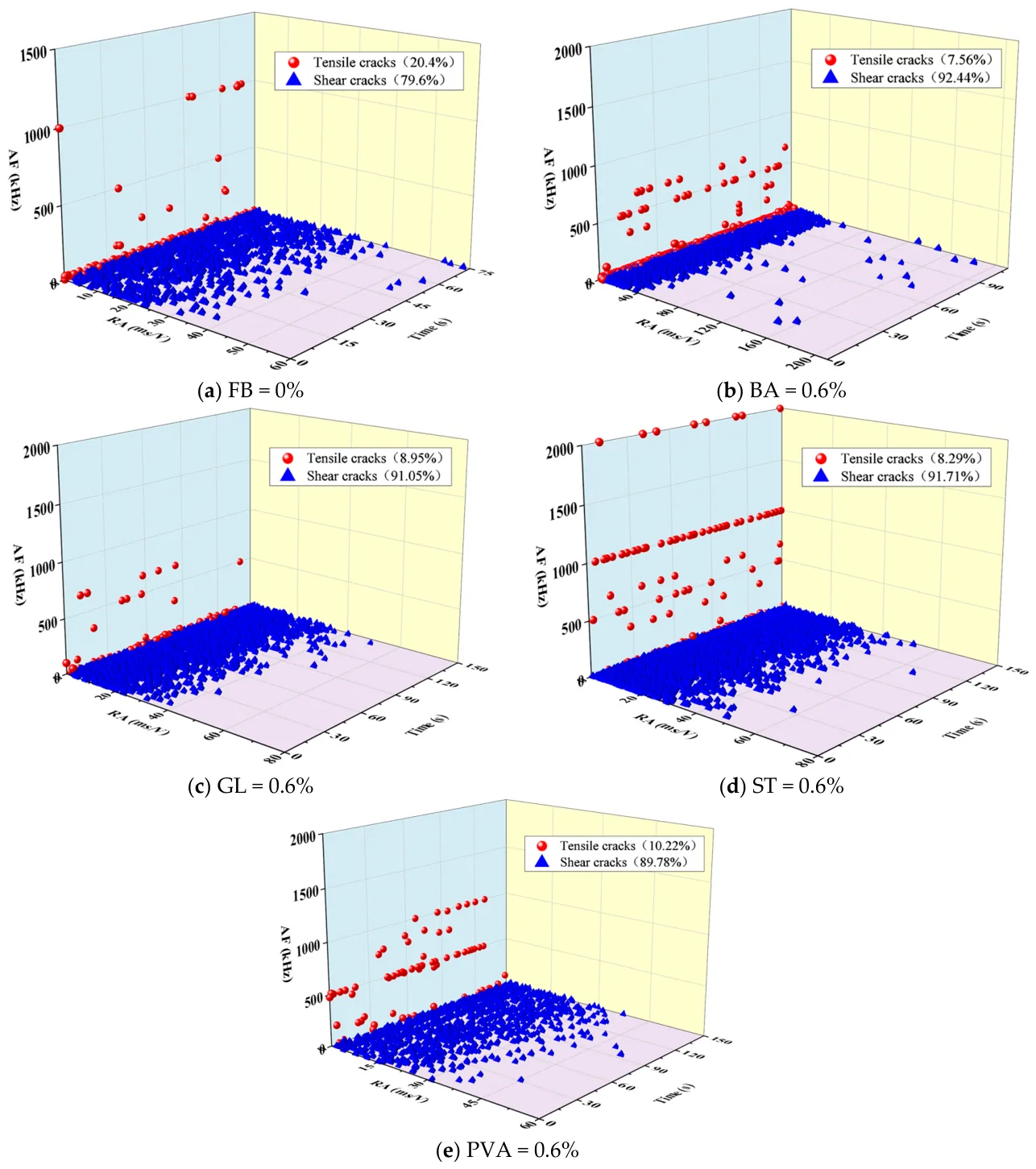
The effect of different fiber types on acoustic emission RA-AF values: (**a**) FB = 0%; (**b**) BA = 0.6%; (**c**) GL = 0.6%; (**d**) ST = 0.6%; (**e**) PVA = 0.6%.

The scatter plot of RA-AF values, as shown in Figure 9, combined with relevant research findings, enables a dynamic classification of tensile and shear cracks [27]. The control group without fiber reinforcement exhibits a high concentration of AE events in the region of high RA and low AF. According to the classification criteria, shear cracks account for as high as 79.6%, indicating that the brittle matrix predominantly undergoes shear sliding and interlayer separation during failure, which aligns with existing research conclusions [28]. However, as illustrated in Figure 9b–e, when fibers of different types are introduced at a content of 0.6%, the AE event distribution becomes even more concentrated in the high RA and low AF region, with the proportion of shear cracks increasing to 92.44%, 91.05%, 91.71%, and 89.78%, representing increases of 16.13%, 14.38%, 15.21%, and 12.78%, respectively. This suggests that the incorporation of fibers has not altered the fundamental brittle failure mode dominated by shear cracking, but rather intensified this trend.

**Figure 10:**
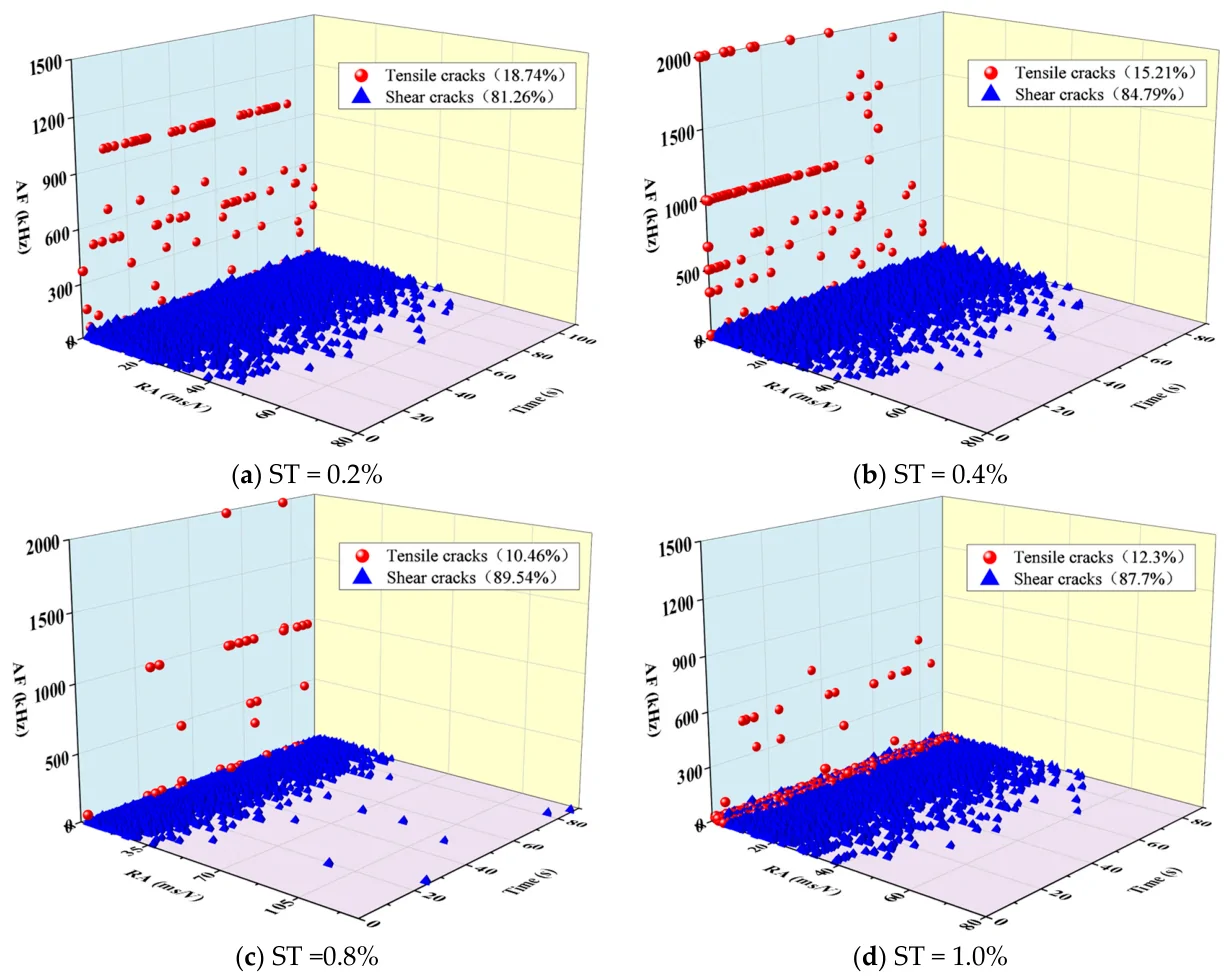
RA-AF values at different contents of copper-coated micro-steel fibers: (**a**) ST = 0.2%; (**b**) ST = 0.4%; (**c**) ST = 0.8%; (**d**) ST = 1.0%.

As presented in Figure 10, the comparison of RA-AF values under different contents of copper-coated micro-steel fibers reveals that when the fiber content is 0.2%, the proportion of shear cracks is 81.26%. Upon increasing the fiber content to 0.4%, this proportion sharply rises to 84.79%. With further increases in fiber content to 0.8% and 1.0%, the proportion of shear cracks remains above 86%, albeit with minor fluctuations. However, at a fiber content of 1.0%, the proportion slightly decreases to 87.7%. This indicates that the introduction of fibers has shifted the damage mode of the material towards shear-dominated cracking, and this effect becomes evident even at relatively low fiber contents, specifically within the range of 0.6% to 0.8%.

### 3.4. Microstructural Characterization of the F-SWG Material Performance

**Figure 11:**
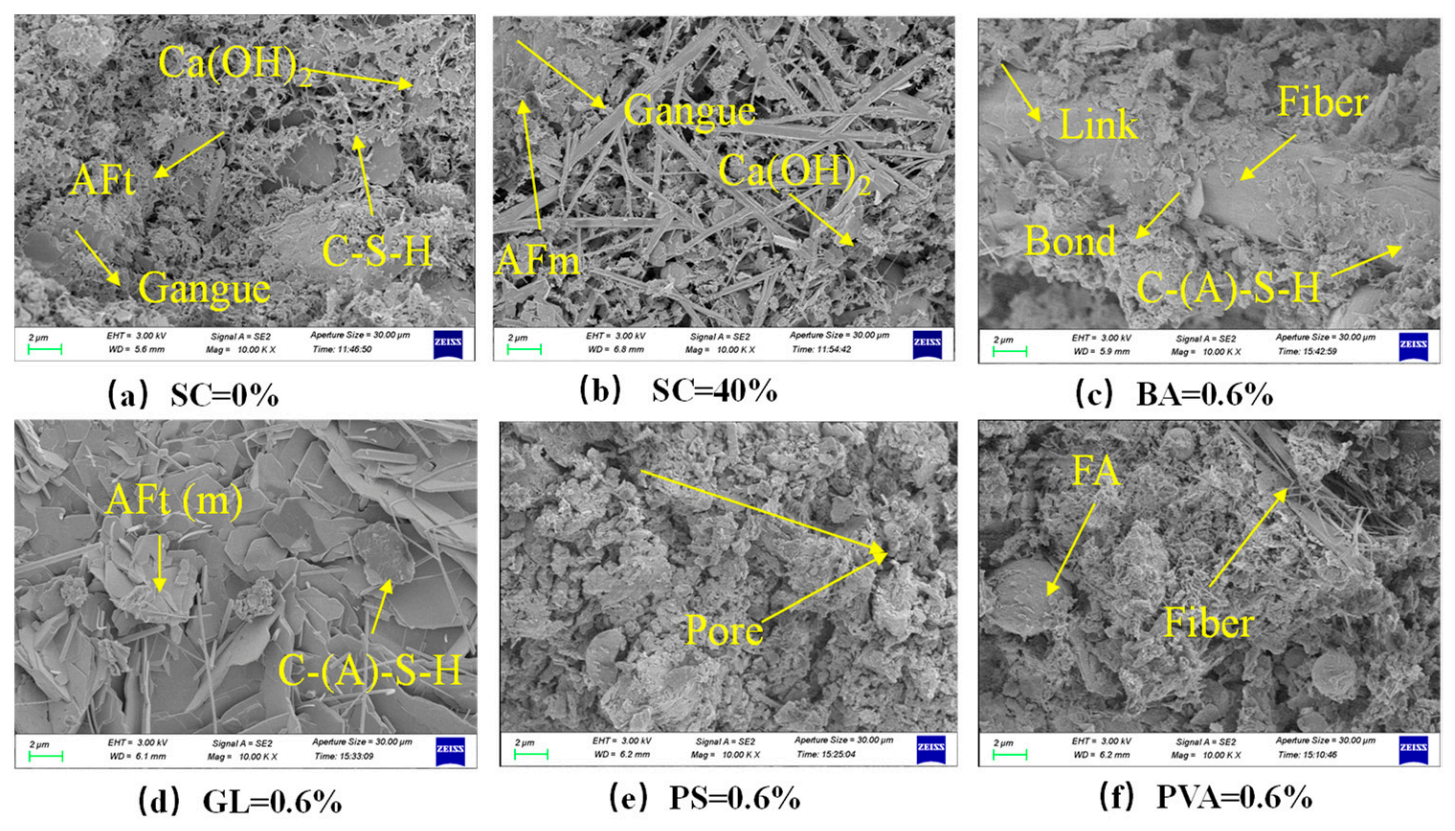
SEM of the F-SWG material: (**a**) SC = 0%; (**b**) SC = 40%; (**c**) BA = 0.6%; (**d**) GL = 0.6%; (**e**) PS = 0.6%; (**f**) PVA = 0.6%.

Figure 11 shows the microstructural images of the sulfoaluminate cement and the corresponding fiber-reinforced specimens. As illustrated in Figure 11a,b, in the case of pure Portland cement systems, a large amount of plate-like calcium hydroxide (CH) and rod-like ettringite (AFt) crystals are closely interwoven and interlocked, forming a rigid framework, which is fully filled by calcium-silicate-hydrate (C-S-H) gel. The resulting microstructure is dense, with fine and uniformly distributed pores. In contrast, when 40% sulfoaluminate cement is incorporated, a significant change in the microstructure is observed. In addition to AFt and CH, a large quantity of hexagonal plate-like monosulfate calcium sulfoaluminate hydrate (AFm) is formed. These hydration products intertwine with unreacted fly ash and coal gangue powder particles, creating a complex three-dimensional network. However, this transformation is accompanied by an increased number of pores on the surface of the material, with some fly ash particles remaining unreacted and clearly identifiable. Overall, the porosity is higher than that of the pure Portland cement system, and the microstructure appears relatively loose.

As shown in Figure 11c–f, under a fiber content of 0.6%, different types of fibers exhibit distinct interfacial morphologies and interaction mechanisms with the cement matrix. The basalt fiber surface displays pronounced pits and a rough texture, with hydration products deeply embedded into the fiber surface. There is nearly no gap between the fiber and the matrix, and the interface is remarkably tight, effectively hindering crack propagation and contributing to excellent flexural strength performance. In contrast, the glass fiber surface is highly smooth and lustrous, with a relatively distinct boundary from the matrix, and small gaps are observable. Hydration products grow around the fiber, forming an independent coating, which results in a limited enhancement in flexural strength. The copper-coated micro-steel fiber is completely enclosed by a thick and dense hydration gel, with the interface between the fiber and the gel being indistinct, giving the appearance of seamless integration. On the other hand, although the surface of the polyvinyl alcohol (PVA) fiber is also smooth, it shows relatively good bonding with the surrounding hydration products, with the fiber contours clearly discernible and the overall microstructure appearing more uniform and compact. In summary, the strong interfacial bonding between the fibers and the surrounding hydration products plays a significant role in bridging cracks during crack propagation, thereby contributing to the macroscopic improvement in flexural performance.

### 3.5. Analysis of the Role of Fibers in Coal-Based Solid Waste Slurry Material

**Figure 12:**
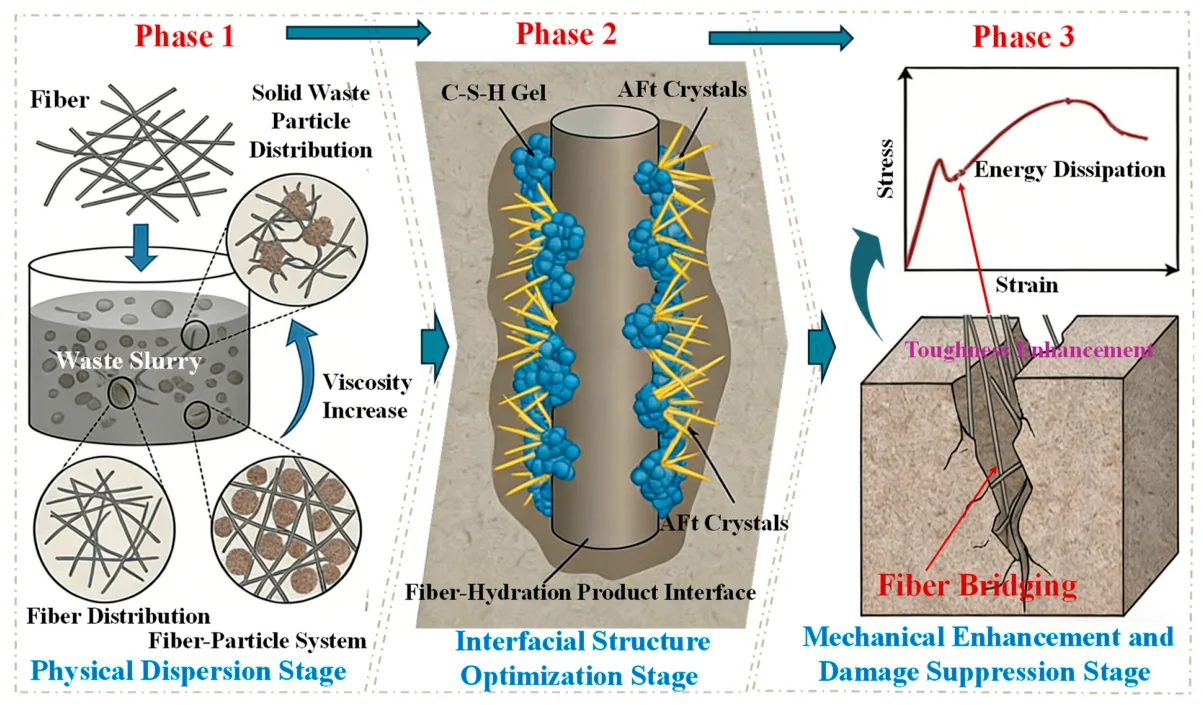
Schematic illustration of the fiber’s role in the coal-based solid waste grouting material at different stages.

As a dispersed solid phase, fibers also function as micro-aggregates within the slurry, exerting a micro-aggregate filling effect. Based on their roles at different stages of the grouting material, this process can be categorized into three phases: the physical dispersion stage, the interfacial structure optimization stage, and the mechanical reinforcement and damage inhibition stage, as illustrated in Figure 12.

(1) Physical Dispersion Stage: During the initial mixing stage of the slurry, fibers are introduced into the solid–liquid two-phase system. Their physical presence increases the solid volume fraction of the slurry, directly altering the free space between particles, which leads to a reduction in slurry flowability and an increase in viscosity. Moreover, due to the van der Waals forces between fibers, they tend to flocculate or agglomerate. This behavior transforms the original suspension dominated by solid particles into a more viscous composite fluid system composed of “fiber-particle” interactions, thereby creating favorable conditions for the effective adsorption and directional growth of hydration products on the fiber surfaces.
(2) Interfacial Structure Optimization Stage: As the hydration reaction progresses, cementitious minerals continuously dissolve, leading to a high concentration of active ions in the pore solution. This promotes the continuous nucleation and growth of highly reactive gel phases (e.g., C-S-H gel, AFt crystals) on the surface. During this stage, owing to the significantly higher surface energy of fibers compared to the matrix, they can adsorb cations such as Ca^2+^ from the solution, thereby inducing the preferential deposition and crystallization of hydration products on the fiber surfaces, forming a “fiber-hydration product” composite interface [29]. The structural density and chemical bonding state of this interfacial region directly determine the interfacial bonding strength between fiber and matrix. The presence of fibers also contributes to the uniform distribution of hydration products and fills the micro-gaps between fibers and matrix, effectively enhancing the micro-aggregate filling effect and fundamentally improving the integrity of the material structure.
(3) Mechanical Reinforcement and Damage Inhibition Stage: When microcracks initiate and propagate within the hardened body due to stress concentration, fibers bridging across the crack surfaces form a “bridging zone.” During crack propagation, the fibers exert closing stresses on the crack walls through their inherent tensile strength and modulus, thereby inhibiting crack opening. More importantly, the fiber bridging mechanism introduces a more complex energy dissipation process into the fracture behavior of the material. This includes fiber elastic elongation, interfacial friction and sliding between fibers and matrix, fiber pull-out, and even brittle fracture of some fibers. Through these mechanisms, the concentrated energy input from the external load is effectively converted into dissipated energy and surface energy via fiber bridging. Consequently, the fracture toughness and critical strain energy release rate of the material are significantly enhanced. In terms of macroscopic performance, this results in a substantial improvement in the flexural strength of the material, meaning that the material not only maintains its load-bearing capacity but also exhibits significant improvements in ductility and toughness.

## 4. Discussion

Previous studies have indicated that the differential toughening efficiency of fibers is fundamentally rooted in the synergy between their physical-mechanical properties and the surrounding cementitious matrix. The following sections will analyze their impact on material performance from the perspectives of bridging efficiency and stress dispersion.

### 4.1. Intrinsic Properties of Fibers and Bridging Efficiency

The primary mechanism of fiber toughening lies in its capacity to transfer stress across the crack interface, i.e., bridging efficiency, which is determined by the fiber’s intrinsic mechanical properties and the interfacial chemical bonding strength between the fiber and the matrix [30]. For instance, basalt fibers, characterized by high stiffness (elastic modulus of 100 GPa) and low elongation (<4%), function as a “rigid skeleton” within the material matrix. When a microcrack tip attempts to propagate under stress, these fibers exert significant counteracting constraints, effectively inhibiting crack extension. More importantly, studies have shown that the abundance of reactive groups on the fiber surface facilitates chemical adsorption with ions such as Ca^2+^ in the cement paste, thereby promoting the oriented growth of C-S-H gel on the fiber surface. This results in the formation of a dense interfacial transition zone [31]. The near-seamless interfacial bonding ensures efficient and damage-free stress transfer from the matrix to the fiber, thus avoiding early failure modes such as interfacial debonding, as illustrated clearly in Figure 11c. In contrast, the inert surface of glass fibers leads to a dominance of physical adhesion at the interface, resulting in relatively low bonding strength. Meanwhile, the low modulus of PVA fibers causes premature deformation under loading, eliminating their ability to support crack propagation. Therefore, the intrinsic toughening effectiveness of fibers is essentially determined by their ability to construct an efficient and stable stress–strain transfer pathway.

### 4.2. Energy Dissipation Mechanism of Fibers in Damage Evolution Paths

The use of DIC and acoustic emission techniques has confirmed that the incorporation of fibers not only enhances the bearing capacity of the material but, more importantly, alters its damage evolution path and failure mode. In the absence of fibers, the damage evolution in the specimens exhibits a highly localized characteristic, with strain rapidly accumulating and leading to the formation of a dominant crack, indicative of brittle fracture, as depicted in Figure 7a. However, the addition of fibers results in a more uniform and diffused strain distribution on the material surface, with high-strain regions being scattered and isolated. This is attributed to the three-dimensional random fiber network formed within the matrix, which redistributes localized and concentrated stresses across a broader area, thereby delaying the formation of macroscopic critical cracks. Additionally, acoustic emission analysis based on RA-AF characterization reveals that the incorporation of fibers enhances the material’s tendency toward shear-dominated failure [15].

## 5. Conclusions

This study systematically investigates the effects of different types and contents of fibers on the flexural performance and damage evolution behavior of grouted materials. The major conclusions are as follows:

(1) Basalt fiber achieves optimal toughening at 0.4% addition, resulting in a 28-day flexural strength of 6.33 MPa, which is 73.9% higher than the control group. Glass fiber also provides significant toughening at 0.8% addition, with a flexural strength increase of 63.18%. The fiber toughening effect exhibits a clear optimal dosage threshold; excessive fiber content leads to agglomeration, which deteriorates mechanical performance.
(2) The introduction of fibers promotes a more uniform and diffuse strain distribution before failure, significantly restraining the propagation of the main crack. The macroscopic failure path becomes more tortuous, and the material transitions from instantaneous brittle fracture to progressive failure. At 0.6% fiber content, the proportion of shear cracks increases by 12.78% to 16.13%.
(3) Microscopic morphology analysis reveals that basalt fibers, with their surface pits, form an almost seamless interface with hydration products, effectively impeding crack propagation. In contrast, glass fibers have smooth surfaces and distinct boundaries with the matrix, leading to weaker interfacial bonding strength, which in turn limits their bridging toughening performance.
(4) Fibers enhance the macroscopic flexural strength of the material through mechanisms such as crack bridging, structural optimization of the interfacial transition zone, and the promotion of shear-dominated damage modes. Overall, the interaction process between fibers and coal-based solid waste can be divided into three distinct stages: dispersion, interfacial structure optimization, and mechanics enhancement with damage inhibition.

## Data Availability

The original contributions presented in this study are included in the article. Further inquiries can be directed to the corresponding author.
